# Lyme Endocarditis as an Emerging Infectious Disease: A Review of the Literature

**DOI:** 10.3389/fmicb.2020.00278

**Published:** 2020-02-26

**Authors:** Aleksandra Nikolić, Darko Boljević, Milovan Bojić, Stefan Veljković, Dragana Vuković, Bianca Paglietti, Jelena Micić, Salvatore Rubino

**Affiliations:** ^1^Faculty of Medicine, University of Belgrade, Belgrade, Serbia; ^2^“Dedinje” Cardiovascular Institute, Belgrade, Serbia; ^3^Institute of Microbiology and Immunology, Faculty of Medicine, University of Belgrade, Belgrade, Serbia; ^4^Department of Biomedical Sciences, University of Sassari, Sassari, Italy; ^5^Clinic for Gynecology and Obstetrics, Clinical Center of Serbia, Belgrade, Serbia

**Keywords:** Lyme disease, Lyme endocarditis, PCR, *Borrelia* spp., valve involvement

## Abstract

Lyme endocarditis is extremely rare manifestation of Lyme disease. The clinical manifestations of Lyme endocarditis are non-specific and can be very challenging diagnosis to make when it is the only manifestation of the disease. Until now, only a few cases where reported. Physicians should keep in mind the possibility of borrelial etiology of endocarditis in endemic areas. Appropriate valve tissue sample should be sent for histopathology, culture, and PCR especially in case of endocarditis of unknown origin PCR on heart valve samples is recommended. With more frequent PCR, *Borrelia* spp. may be increasingly found as a cause of infective endocarditis. Prompt diagnosis and treatment of Lyme carditis may prevent surgical treatment and pacemaker implantations. Due to climate change and global warming Lyme disease is a growing problem. Rising number of Lyme disease cases we can expect and rising number of Lyme endocarditis.

## Lyme Endocarditis Highlights

– Lyme endocarditis is a rare manifestation of Lyme disease
– Manifestations of Lyme endocarditis are non-specific, and diagnosis can be challenging
– In case of endocarditis of unknown origin, Polymerase Chain Reaction (PCR) of heart valve samples is recommended
– If routine analyses do not reveal a pathogen agent, the physician should think about Lyme endocarditis
– When a cardiac surgeon, during surgery, suspects infective endocarditis, a tissue sample should be taken for histopathology, culture, and PCR
– Prompt diagnosis and treatment of Lyme carditis may avoid surgical treatment and pacemaker implantation
– Due to climate change, we can expect more cases of Lyme carditis with involvement of heart valve

## Introduction

Almost 45 years after its first recognition, Lyme disease (LD) has recently become a huge and growing problem, both in Europe and the United States (Lindgren and Jaenson, 2006). Due to global climate changes, LD is emerging as a threat to public health, easy spreading rapidly into new territories with a lack of a prevention method. The vector that carries the infective agent can be found in places where it has never been found before (Estrada-Peña et al., 2018), and these tick distribution and density changes have been shown to be related to changes in climate (Liang and Gong, 2017). If it is not immediately recognized and treated, it can be a life-threatening disease with multiple cardiac and neurological manifestations (Kannangara et al., 2019).

Lyme carditis (LC) is a rare manifestation of LD that includes: heart conduction abnormalities, myocarditis, pericarditis, endocarditis, pancarditis, arrhythmias, dilatative cardiomyopathy and congestive heart failure, myocardial infarction, and coronary aneurysms (Hidri et al., 2012; Kostić et al., 2017). Lyme endocarditis (LE), one possible manifestation of LC, is rare and has been the subject of case reports. Due to climate change, an increased incidence rate of LC with involvement of heart valve can be expected (Lindgren and Jaenson, 2006; Liang and Gong, 2017; Estrada-Peña et al., 2018; Kannangara et al., 2019).

## Epidemiology

Lyme disease is endemic and the most common vector-borne bacterial disease transmitted to humans in North America, Europe, and Asia. In the United States, around 60,000 cases were reported in 2017, according to the Centers for Disease Control and Prevention (CDC), an increase of more than 20% over 2016 (Centers for Disease Control and Prevention [CDC], 2018). In 2015, LD was the sixth most common nationally notifiable disease in the United States. In the states where LD is most common, the average incidence is 39.5 cases per 100,000 persons (Northeast, mid-Atlantic, and upper Midwest of the United States) (Bacon et al., 2008). It seems that this number is underestimated and that the actual incidence of LD could be as much as 10 times higher than the CDC data indicate. This is a result of inadequate reporting, misdiagnosis, and the fact that physicians tend to underreport reportable diseases (Adams et al., 2015).

Estimation from available national data suggests that there are about 85,000 cases per year in Europe where most LD is reported by Scandinavian countries, Germany, Austria, and Slovenia (Lindgren and Jaenson, 2006). Smith and Takkinen (2006) showed that the estimated incidence of LD was as high as 206 cases per 100,000 population in Slovenia and 135 cases per 100,000 population in Austria, which are among the highest reported rates in Europe. Increases in prevalence have also been observed in Sweden, Germany, Czechia, Norway, and Finland (Jaenson and Lindgren, 2011; Heinz et al., 2015; Semenza and Suk, 2017). In Asia, *Borrelia burgdorferi* infection has been reported in countries including China, Korea, Japan, Indonesia, and Nepal and in eastern Turkey (Jaenson and Lindgren, 2011; Heinz et al., 2015). Beside the above-mentioned areas, cases were reported in more tropical locales, and LD may exist in Australia (Dehhaghi et al., 2019).

## Cardiac Manifestation of Lyme Disease and Valve Involvement

Lyme carditis is rare, representing only 0.3–4% of cases in Europe (Hidri et al., 2012). In the United States, between 4 and 10% of patients who do not undergo treatment of LD develop carditis (Paim et al., 2018). LC is associated with acute-onset atrioventricular blocks (I–III), which are the most common feature of LC, arrhythmias and myocarditis or pericarditis, and pericardial effusion, while the chronic stage includes dilated cardiomyopathy (Palecek et al., 2010; Hidri et al., 2012). In a review of 84 patients who had LC, 69% reported palpitations, 19% had conduction abnormalities, 10% had myocarditis, and 5% had left ventricular systolic dysfunction (Paim et al., 2018).

Valvular involvement, as a manifestation of LC, is extremely rare and is the subject of case reports (Hidri et al., 2012; Paim et al., 2018). To date, seven cases in the adult population have been reported and one in the pediatric population. It is important to know that complete conduction recovery with antibiotic treatment occurs in more than 90% of LC cases (Kostić et al., 2017). In areas where LD is endemic, the evaluation of acute-onset cardiac symptoms, with evidence of conduction disease or valvular pathology, should lead to a work-up for LC (Palecek et al., 2010; Kostić et al., 2017; Paim et al., 2018). Prompt diagnosis and treatment of LC may prevent unnecessary surgical treatment, pacemaker implantation, or treatment of heart failure (Palecek et al., 2010; Hidri et al., 2012; Paim et al., 2018).

## Pathoimmunology

*Borrelia burgdorferi* is a highly invasive spirochete that produces adhesions. Via these proteins, *B. burgdorferi* adheres to endothelial cells and to components of the extracellular matrix. By changing of its surface, *B. burgdorferi* modifies immunological response and decreases the phogocytosis of the infected organism (Zajkowska et al., 2000; Zajkowska and Hermanowska–Szpakowicz, 2002; Raveche et al., 2005). Wasiluk et al., in their paper, stated that, in resistant chronic LD, autoimmune mechanisms play a role in persistent disease (Wasiluk et al., 2011). *B. burgdorferi* displays tropism to heart connective tissue, synovial membrane, ligaments, tendon attachments, and vascular endothelium, where it makes molecular changes (dominantly in Lyme arthritis and LD) (Froude et al., 1989; Grzesik et al., 2004; Raveche et al., 2005; Petzke and Schwartz, 2015). There is a belief that autoimmunity and genetic predisposition may play important roles in the inflammation process. One of the mechanisms of autoimmunity is molecular mimicry. By aggregation with fibroblasts and tissue proteins, *B. burgdorferi* disturbs the secretion of cytokines and antibodies, but it can directly attack and destroy T and B lymphocytes as well (Zajkowska et al., 2000; Raveche et al., 2005). This spirochete activates chemotactic factors by induction of interleukins (IL) IL1, IL6, IL8, and IL10, mediators of inflammation, and immunological complexes and activates the complement system (Steere et al., 2001; Tuchocka, 2002; Raveche et al., 2005; Wasiluk et al., 2011).

## Lyme Endocarditis: a Review of the Literature

A computerized literature search was conducted using the PubMed databases for relevant articles on LE published in English from 1977 to July of 2019. A possible connection between LD and valve involvement in a 56-year-old male was described in 1993 by Anish. The diagnosis was based on clinical findings: aortic valvular vegetation revealed by transesophageal echocardiography, positive *Borrelia* spp. serology, and prompt improvement after ceftriaxone therapy. However, neither histopathologic nor molecular screening of valvular tissue was performed in order to confirm the suspicions of LE (Anish, 1993). Canver et al. described a case of fulminant LE with involvement of mitral valve in a *B. burgdorferi* seropositive patient, but again, without valve tissue analysis for confirmation (Canver et al., 2000). *Borrelia bissettii was* detected in the aortic valve tissue of a patient with endocarditis and aortic stenosis in the Czechia. Molecular analysis of a valve sample confirmed the presence of *B. bissettii* DNA (Rudenko et al., 2008).

Hidri et al. (2012) described the first case of *Borrelia afzelii* LE, in a 61-year-old man living in an endemic area of France. They reported a case of endocarditis of mitral valve. The diagnosis was confirmed by detection of *B. afzelii* DNA by specific real-time Polymerase Chain Reaction (PCR). The patient was scheduled for mitral valve replacement due to mitral regurgitation caused by mitral valve prolapse and rope rupture. During surgery, the macroscopic analysis of the mitral valve, showing prolapse of the posterior and perforation of the anterior valve, suggested endocarditis. All routinely performed microbiological analysis was negative, but microscopic analysis revealed intracellular microorganisms that were ultimately confirmed as *B. afzelii.* This case emphasized the need to perform PCR on heart valve samples in the case of endocarditis of unknown origin and to have in mind the possibility of borrelial etiology in endemic areas.

The first case of LE confirmed by molecular diagnostics in the United States was reported by Paim et al. (2018) in a 68-year-old male who presented with heart failure with suspected community-acquired pneumonia. Transesophageal echocardiogram revealed severe mitral regurgitation due to aneurismal dilatation of the anterior mitral leaflet with a perforation. These findings suggested infectious etiology. The patient reported LD 8 years previously, which was treated with two sequential courses of doxycycline. The results of serological testing were negative, including four blood cultures as well as virus testing and PCR of *Tropheryma whippelii* and *Borrelia* spp. Results of the 16S rRNA PCR and sequencing performed on resected mitral valve tissue were positive for *B. burgdorferi*. Diagnosis of LE can be very challenging due to an inability to grow the organism in culture, and serologic testing cannot distinguish current from prior infection, especially when it is the only manifestation of the disease (Paim et al., 2018).

Authors from Cleveland reported a case of LE confirmed by PCR without prior clinical manifestations of LD. They described a case of a 65-year-old female with mitral regurgitation due to myxomatous mitral valve degeneration and valve prolapse who underwent mitral valve repair. She denied tick bites or annular rash. During the operation, the surgeon suspected an infective rather than degenerative etiology due to anterior leaflet scarring and destruction over the A2 area, with thickened chords. All tissue cultures were negative. DNA of *B. burgdorferi* was identified by 16S ribosomal ribonucleic acid sequencing. This case suggests that when the cardiac surgeon suspects on infective pathology, tissue samples should be sent for culture, histopathology, and PCR analysis (Haddad et al., 2019).

Cardiac manifestations of LD typically include the atrioventricular conduction system, rarely heart valves. Patel and Schachne (2017) reported a case of a 59-year-old male with involvement of both the electrical conduction system and the mitral valve. LD was diagnosed on the basis of elevated immunoglobulins IgA, IgM, and IgG to *B. burgdorferi*. The authors revealed that mitral regurgitation was likely to be chronic rather than acute due to the echo parameters. Complete recovery of the conduction system after antibiotic treatment was noticed in this case, and stress echocardiography showed reduced mitral regurgitation. Their conclusion was that local invasion of pathogen and macrophage caused leaflet edema. Histopathology was not done, and they speculated whether it was possible that local myocardial inflammation had worsened the regurgitation of a chronically diseased valve. Finally, in areas endemic for LD, the evaluation of acute-onset cardiac symptoms, especially with evidence of conduction disease or valvular pathology, physicians should think of LC and LE. Prompt diagnosis and treatment of LD and LE may prevent surgical treatment and pacemaker implantation (Patel and Schachne, 2017).

The only LD case in children with suspected involvement of mitral valve was reported by Kameda et al. (2012) They described an unusual manifestation of LD in a 7-year-old girl with Lyme neuroborreliosis with meningoradiculitis and involvement of mitral valve that was discovered due to a heart murmur. She was treated by cefotaxime for 4 weeks. After one year, echo showed a normal mitral valve with trivial mitral regurgitation. In children, LE is extremely rare, and this is the only case in the literature with suspected valve involvement in a child. Despite improvement after therapy, there was no evidence from histopathology or PCR of the valve tissue and no definitive diagnosis of LE (Kameda et al., 2012). A summary of the case reports is given in Table 1.

**TABLE 1:** Summary of Case Reports of Lyme Endocarditis.

**References**	**Valve type**	**Serologic test (blood)**	**Valve histopathology**	**Valve culture**	**Molecular testing**	**Treatment**
Anish, 1993	Aortic	EIA 1.10 and repeat 1 week later 1.22 Western blot IgG P-41 band positive	Not performed	Not performed	Not performed	Ceftriaxone 2 g IV daily for 2 weeks, then doxycycline 100 mg po b.i.d for 30 days
Canver et al., 2000	Mitral	ELISA reactive (IgG and IgM). Immunoblot: 6 antigenic bands in IgG probe	Myxoid degeneration with infiltration of lymphocytes No evidence of fibrinoid exudate or Aschoff bodies	Not performed	Not performed	Not specified
Rudenko et al., 2008	Aortic	ELISA reactive (IgG) Western blot positive	Highly calcified dissected cardiac valve	Negative for aerobic and anaerobic microorganisms. Negative for spirochetes	PCR amplification: 99% identity to the *flagellin* gene of *Borrelia bissettii* strain	Antimicrobial therapy, not specified
Hidri et al., 2012	Mitral	ELISA reactive (IgG) Immunoblot: 6 antigenic bands in IgG probe	Endocarditis with foamy macrophages suggestive of intracellular microorganisms; Gram, PAS, and Giemsa stains were negative. Wharton-Starry stain showed only scarce curved rods, which had a morphology that was not specific to spirochetes.	Not specified	Universal PCR targeting 16S RNA-encoding DNA identified the genus *Borrelia.* Real time DNA amplification identified *B. afzelii*	Valve replacement (IV gentamicin and amoxicillin for 2 weeks, followed by 4 weeks of oral amoxicillin
Kameda et al., 2012	Mitral	ELISA reactive (IgG and IgM) Immunoblot: positive (data not shown)	Not performed	Not performed	Not performed	Cefotaxime (200 mg/kg/day) for 14 days
Patel and Schachne, 2017	Mitral	EIA) detected significant levels of IgG (24.3; ref range <1), IgM (9.6; ref range <1), and IgA (>9.9; ref range <1) to B. burgdorferi.	Not performed	Not performed	Not performed	Ceftriaxone IV in hospital followed by oral doxycycline at home
Paim et al., 2018.	Mitral	Results of serologic blood testing for *Bartonella* spp., *Coxiella burnetii*, *Chlamydia* spp., *Legionella pneumophila*, *Blastomyces dermatitidis*, *Coccidioides* spp., *Histoplasma capsulatum*, and HIV infection were negative. Serum cryptococcal and urinary *Histoplasma* antigens were both negative.	Not performed	Histopathology showed active native valve endocarditis with no microorganisms identified by Gram, Gomori methenamine silver, and periodic acid–Schiff-diastase (PAS-D), and Steiner stains.	PCR testing from the blood for *Tropheryma whippelii* and *Borrelia* spp. were negative. Results of the 16S rRNA PCR and sequencing on resected mitral valve tissue were positive for *B. burgdorferi*.	Ceftriaxone for 6 weeks (not specified)
Haddad et al., 2019	Mitral	Blood cultures, serology for *Bartonella* and *Coxiella* were unrevealing. IgM and IgG were positive by enzyme immunoassay as well as Western blot (2 IgM bands and 10 IgG bands)	Negative	Cultures were negative and histopathological evaluation of the submitted limited valve tissue was non-diagnostic.	16S Ribosomal ribonucleic acid (rRNA) sequencing identified DNA of *Borrelia burgdorferi*	The patient was treated with ceftriaxone 2 g IV q 24 h for 6 weeks

Lyme endocarditis is a rare condition, but new cases can be expected due to climate change. Global warming, as one of the components of climate change, has increased significantly in recent years, and it has had a huge impact on human health (Watts et al., 2015). The effect of global warming on human health is divided into two categories: a direct effect on disease, such as heat shock and increased mortality in the population with other diseases, and an indirect effect on diseases such as infectious diseases and allergies. Heatwaves, storms, drought, and floods result in a shift in the distribution of pathogens and vectors, which consequently results in a shift in the distribution of human infectious disease (Kuhn et al., 2005; Tian et al., 2015).

Tick-born disease and the transmission of disease-causing agents are significantly influenced by weather and climate as well, which has an indirect effect on humans (IPCC, 2013). Milder winters and warmer falls and springs may enable the extension of Lyme borreliosis to higher altitudes and latitudes, predominantly in the north of Europe (Semenza and Menne, 2009). Thus, the level of influence depends on the kind of vector (Confalonieri et al., 2007; Kurane, 2010).

## Diagnosis

Lyme disease, and especially LE, can be very challenging to diagnose. The most specific (100%) examination is culture of *Borrelia* spp. This test provides information on active infection, enables the investigation of the structural, molecular, antigenic, and pathogenic properties of the antigen, and can distinguish live from dead organisms (Reed, 2002; Aguero-Rosenfeld et al., 2005; Murray and Shapiro, 2010). But, there are limitations to this exam. The main limitation is the time needed for the culture to reveal its results (up to 12 weeks for it to be considered negative). Other issues are the low sensitivity of the method in all other than the cutaneous manifestation of the disease, its inapplicability for diagnosis in antibiotic-treated patients, the need for special media, and its expense (Karlsson et al., 1990; Nadelman et al., 1990).

Serological examination is widely used and is available in the clinical setting. Frequently used assays are enzyme-linked immunosorbent assay (ELISA), immunofluorescence assays, and Western blotting (Tugwell, 1997; Schutzer et al., 2018), but false-negative and false-positive results may occur (Schutzer et al., 2018). ELISA is recommended as the initial serological examination. Tests are objective, fast, and easy to perform and are suitable for the diagnosis of other forms of LD beside cutaneous. This is the reason why, in the United States, a two-step protocol for the evaluation of *B. burgdorferi* antibodies in sera has been recommended (Miller et al., 2018). In both ELISA and Immunoblot assays, the antigens used should detect both IgM and IgG antibodies. Immunoblot should have a high specificity of at least 95% (Miller et al., 2018; Schutzer et al., 2018). In the early stages of the disease, serological tests may reveal false-negative results in a high percentage. False-positive results can be seen in mononucleosis, autoimmune states and *Treponema pallidum* infection. Western blot is more sensitive and specific than ELISA, and it is recommended as a second step in diagnosis and for confirmation of ELISA findings (Miller et al., 2018).

Polymerase Chain Reaction can be very helpful, since it detects the genetic material of *Borreliae* sp. directly in multiple tissues and provides molecular identification and antimicrobial therapy does not affect the results (Rijpkema et al., 1997). Transvenous endomyocardial biopsy can be indicative of LD. The band-like infiltrate is strongly suggestive, and it can be seen even if the quality of the specimen is limited (Marques, 2015). Its intrinsic limitations, which include sampling error, the necessity that the patient undergo an invasive procedure to obtain appropriate tissue, and the variability of interpretation, restrain its use in clinical practice (Reed, 2002; Aguero-Rosenfeld et al., 2005).

## Treatment

Antibiotic therapy in the early stages of LD prevents or attenuates later complications of the disease (Sangha et al., 1998). Antibiotic regimens for the treatment of LD include amoxicillin 500 mg orally three or four times daily for 30 days, doxycycline 100 mg orally twice daily for 30 days, and ceftriaxone 2 g intravenously daily for 2 to 4 weeks (Fish et al., 2008). Cefotaxime 3 g intravenously twice daily for 2 to 4 weeks is reportedly as effective as ceftriaxone in patients who have other late manifestations of LD (50, 51). Patients who have minor cardiac involvement (e.g., prolongation of the PR interval of no more than 0.30 s) and no other symptoms should receive oral antibiotic therapy with amoxicillin or doxycycline as for early disease. Patients who have more severe LC, should be admitted to hospital and administered intravenous ceftriaxone or high-dose penicillin G. As has been mentioned, complete heart block generally resolves within 1 week, with resolution of lesser conduction disturbances within 6 weeks (Olson et al., 1986; McAlister et al., 1989; Fish et al., 2008). A summary of LE treatment from limited cases in the literature is given in Table 1.

## Summary and Conclusion

Lyme endocarditis is an extremely rare manifestation of LD. The clinical manifestations of LE are non-specific, and it can be a very challenging diagnosis to make when it is the only manifestation of the disease. Until now, only a few cases have been reported. In case of endocarditis of unknown origin, PCR on heart valve samples is recommended. Physicians should keep in mind the possibility of a borrelial etiology of endocarditis in endemic areas. An appropriate valve tissue sample should be sent for histopathology, culture, and PCR when a cardiac surgeon suspects infective endocarditis during an operation. The use of PCR is crucial to detect and identify the causative organism in infective endocarditis, especially in those with negative blood and tissue cultures. With more frequent PCR, *Borrelia* sp. may be increasingly found to be the cause of infective endocarditis. Prompt diagnosis and treatment of LE and LC may allow surgical treatment and pacemaker implantation to be avoided. Due to climate change and global warming, LD is a growing problem, and, due to the rising number of LD cases, we can expect a rising number of LE cases as well.

## Author Contributions

AN, DB, and MB conceptualized this manuscript, collected the data, and drafted the first manuscript. BP and SR revised final revision of the manuscript. All authors provided critical feedback and contributed to the manuscript.

## Conflict of Interest

The authors declare that the research was conducted in the absence of any commercial or financial relationships that could be construed as a potential conflict of interest.

## References

[B1] Adams D. Fullerton K. Jajosky R. Sharp P. Onweh D. Schley A. (2015). Summary of notifiable infectious diseases and conditions – United States, 2013. *MMWR Morb. Mortal. Wkly. Rep.* 62 1–122. 10.15585/mmwr.mm6253a1 26492038

[B2] Aguero-Rosenfeld M. Wang G. Schwartz I. Wormser G. (2005). Diagnosis of Lyme borreliosis. *Clin. Microbiol. Rev.* 18 484–509. 10.1128/cmr.18.3.484-509.2005 16020686 PMC1195970

[B3] Anish S. A. (1993). Case report: possible Lyme endocarditis. *N. J. Med*. 90 599–601. 8414207

[B4] Bacon R. M. Kugeler K. J. Mead P. S. (2008). Surveillance for Lyme disease-United States, 1992-2006. *MMWR Surveill. Summ.* 57 1–9. 18830214

[B5] Canver C. C. Chanda J. DeBellis D. M. Kelley J. M. (2000). Possible relationship between degenerative cardiac valvular pathology and Lyme disease. *Ann. Thorac. Surg.* 70 283–285. 10.1016/s0003-4975(00)01452-1 10921726

[B6] Centers for Disease Control and Prevention [CDC] (2018). *Lyme Disease: Data and Surveillance.* Available online at: http://www.cdc.gov/lyme/stats/index.html?s_cid=cs_281 (accessed October, 2019).

[B7] Confalonieri U. Menne B. Akhtar P. (2007). “Human health. Climate change: impacts, adaptation and vulnerability,” in *Contribution of Working Group II to the Fourth Assessment Report of the Intergovernmental Panel on Climate Change*, eds Parry M. L. Canziani O. F. Palutikof J. P. (Cambridge: Cambridge University Press), 391–431.

[B8] Dehhaghi M. Kazemi Shariat Panahi H. Holmes E. Hudson B. Schloeffel R. Guillemin G. (2019). Human tick-borne diseases in Australia. *Front. Cell. Infect. Microbiol*. 9:3 10.3389/fcimb.2019.00003 PMC6360175 30746341

[B9] Estrada-Peña A. Cutler S. Potkonjak A. Vassier-Tussaut M. Van Bortel W. Zeller H. (2018). An updated meta-analysis of the distribution and prevalence of *Borrelia burgdorferi* s.l. in ticks in Europe. *Int. J. Health Geogr.* 17:41. 10.1186/s12942-018-0163-7 30514310 PMC6319795

[B10] Fish A. Pride Y. Pinto D. (2008). Lyme carditis. *Infect. Dis. Clin. North Am*. 22 275–288. 10.1016/j.idc.2007.12.008 18452801

[B11] Froude J. Gibofsky A. Buskirk D. R. Khanna A. Zabriskie J. B. (1989). Cross-reactivity between streptococcus and human tissue: a model of molecular mimicry and autoimmunity. *Curr. Top. Microbiol. Immunol.* 145 5–26. 10.1007/978-3-642-74594-2_2 2680297

[B12] Grzesik P. Oczko-Grzesik B. Kepa L. (2004). *Borrelia burgdorferi* pathogenesis and the immune response. *Przegl. Epidemiol.* 58 589–596. 15810500

[B13] Haddad O. Gillinov M. Fraser T. Shrestha N. Pettersson G. (2019). Mitral valve endocarditis: a rare manifestation of Lyme disease. *Ann. Thorac. Surg.* 108 e85–e86. 10.1016/j.athoracsur.2018.12.046 30690018

[B14] Heinz F. Stiasny K. Holzmann H. Kundi M. Sixl W. Wenk M. (2015). Emergence of tick-borne encephalitis in new endemic areas in Austria: 42 years of surveillance. *Euro Surveill.* 20 9–16. 10.2807/1560-7917.es2015.20.13.21077 25860391

[B15] Hidri N. Barraud O. de Martino S. Garnier F. Paraf F. Martin C. (2012). Lyme endocarditis. *Clin. Microbiol. Infect.* 18 E531–E532. 10.1111/1469-0691.12016 23043635

[B16] IPCC, (2013).“Summary for policymakers,” in *Climate Change 2013: The Physical Science Basis. Contribution of Working Group I to the Fifth Assessment Report of the Intergovernmental Panel on Climate Change*, eds Stocker T. F. Qin D. Plattner G.-K. Tignor M. Allen S. K. Boschung J. (Cambridge: Cambridge University Press).

[B17] Jaenson T. G. T. Lindgren E. (2011). The range of *Ixodes ricinus* and therisk of contracting Lyme borreliosis will increase northwards when the vegetation period becomes longer. *Ticks Tick Borne Dis.* 2 44–49. 10.1016/j.ttbdis.2010.10.006 21771536

[B18] Kameda G. Vieker S. Hartmann J. Niehues T. Längler A. (2012). Diastolic heart murmur, nocturnal back pain, and lumbar rigidity in a 7-year girl: an unusual manifestation of Lyme disease in childhood. *Case Rep.* 2012:976961. 10.1155/2012/976961 23056982 PMC3465881

[B19] Kannangara D. Sidra S. Pritiben P. (2019). First case report of inducible heart block in Lyme disease and an update of Lyme carditis. *BMC Infect. Dis.* 19:428. 10.1186/s12879-019-4025-0 31096922 PMC6524294

[B20] Karlsson M. Hovind-Hougen K. Svenungsson B. Stiernstedt G. (1990). Cultivation and characterization of spirochetes from cerebrospinal fluid of patients with Lyme borreliosis. *J. Clin. Microbiol.* 28 473–479. 10.1128/jcm.28.3.473-479.1990 2324275 PMC269647

[B21] Kostić T. Momèilović S. Perišić Z. Apostolović S. Cvetković J. Jovanović A. (2017). Manifestations of Lyme carditis. *Int. J. Cardiol.* 232 24–32. 10.1016/j.ijcard.2016.12.169 28082088

[B22] Kuhn K. Campbell-Lendrum D. Haines A. Cox J. (2005). *Using Climate to Predict Infectious Disease Epidemics.* Geneva: World Health Organization.

[B23] Kurane I. (2010). The effect of global warming on infectious diseases. *Osong Public Health Res. Perspect*. 1 4–9. 10.1016/j.phrp.2010.12.004 24159433 PMC3766891

[B24] Liang L. Gong P. (2017). Climate change and human infectious diseases: a synthesis of research findings from global and spatio-temporal perspectives. *Environ. Int.* 103 99–108. 10.1016/j.envint.2017.03.011 28342661

[B25] Lindgren E. Jaenson T. G. T. (2006). *Lyme Borreliosis in Europe: Influences of Climate and Climate Change, Epidemiology, Ecology and Adaptation Measures.* Copenhagen: WHO Regional Office for Europe.

[B26] Marques A. (2015). Laboratory diagnosis of Lyme disease. *Infect. Dis. Clin. North Am.* 29 295–307. 10.1016/j.idc.2015.02.005 25999225 PMC4441761

[B27] McAlister H. F. Klementowicz P. T. Andrews C. Fisher J. D. Feld M. Furman S. (1989). Lyme carditis: an important cause of reversible heart block. *Ann. Intern. Med.* 110 339–345. 2644885 10.7326/0003-4819-110-5-339

[B28] Miller J. Binnicker M. Campbell S. Carroll K. Chapin K. Gilligan P. (2018). A guide to utilization of the microbiology laboratory for diagnosis of infectious diseases: 2018 update by the infectious diseases society of America and the American Society for Microbiologya. *Clin. Infect. Dis.* 67 e1–e94. 10.1093/cid/ciy381 29955859 PMC7108105

[B29] Murray T. Shapiro E. (2010). Lyme disease. *Clin. Lab. Med.* 30 311–328. 10.1016/j.cll.2010.01.003 20513553 PMC3652387

[B30] Nadelman R. Pavia C. Magnarelli L. Wormser G. (1990). Isolation of *Borrelia burgdorferi* from the blood of seven patients with Lyme disease. *Am. J. Med.* 88 21–26. 10.1016/0002-9343(90)90122-t 2294761

[B31] Olson L. J. Okafor E. C. Clements I. P. (1986). Cardiac involvement in Lyme disease: manifestations and management. *Mayo Clin. Proc.* 61 745–749. 10.1016/s0025-6196(12)62775-x 3747616

[B32] Paim A. Baddour L. Pritt B. Schuetz A. Wilson J. (2018). Lyme endocarditis. *Am. J. Med.* 131 1126–1129. 10.1016/j.amjmed.2018.02.032 29605416

[B33] Palecek T. Kuchynka P. Hulinska D. Schramlova J. Hrbackova H. Vitkova I. (2010). Presence of *Borrelia burgdorferi* in endomyocardial biopsies in patients with new-onset unexplained dilated cardiomyopathy. *Med. Microbiol. Immunol.* 199 139–143. 10.1007/s00430-009-0141-146 20052487

[B34] Patel L. Schachne J. (2017). Lyme carditis: a case involving the conduction system and mitral valve. *R. I. Med. J. (2013)* 100 17–20. 28146594

[B35] Petzke M. Schwartz I. (2015). *Borrelia burgdorferi* pathogenesis and the immune response. *Clin. Lab. Med.* 35 745–764. 10.1016/j.cll.2015.07.004 26593255

[B36] Raveche E. Schutzer S. Fernandes H. Bateman H. McCarthy B. Nickell S. (2005). Evidence of *Borrelia* autoimmunity-induced component of Lyme carditis and arthritis. *J. Clin. Microbiol.* 43 850–856. 10.1128/jcm.43.2.850-856.2005 15695691 PMC548028

[B37] Reed K. (2002). Laboratory testing for Lyme disease: possibilities and practicalities. *J. Clin. Microbiol.* 40 319–324. 10.1128/jcm.40.2.319-324.2002 11825936 PMC153420

[B38] Rijpkema S. Tazelaar D. Molkenboer M. Noordhoek G. Plantinga G. Schouls L. (1997). Detection of *Borrelia afzelii*, *Borrelia burgdorferi* sensu stricto, *Borrelia garinii* and group VS116 by PCR in skin biopsies of patients with erythema migrans and acrodermatitis chronica atrophicans. *Clin. Microbiol. Infect.* 3 109–116. 10.1111/j.1469-0691.1997.tb00259 11864084

[B39] Rudenko N. Golovchenko M. Mokracek A. Piskunova N. Ruzek D. Mallatova N. (2008). Detection of *Borrelia bissettii* in cardiac valve tissue of a patient with endocarditis and aortic valve stenosis in the Czech Republic. *J. Clin. Microbiol.* 46 3540–3543. 10.1128/jcm.01032-08 18650352 PMC2566110

[B40] Sangha O. Phillips C. B. Fleischmann K. E. (1998). Lack of cardiac manifestations among patients with previously treated Lyme disease. *Ann. Intern. Med.* 128 346–353. 9490594 10.7326/0003-4819-128-5-199803010-00002

[B41] Schutzer S. Body B. Boyle J. Branson B. Dattwyler R. Fikrig E. (2018). Direct diagnostic tests for Lyme disease. *Clin. Infect. Dis.* 68 1052–1057. 10.1093/cid/ciy614 30307486 PMC6399434

[B42] Semenza J. Suk J. (2017). Vector-borne diseases and climate change: a European perspective. *FEMS Microbiol. Lett.* 365:fnx244 10.1093/femsle/fnx244 PMC5812531 29149298

[B43] Semenza J. C. Menne B. (2009). Climate change and infectious diseases in Europe. *Lancet Infect. Dis.* 9 365–375. 10.1016/S1473-3099(09)70104-5 19467476

[B44] Smith R. Takkinen J. (2006). Lyme borreliosis: Europe-wide coordinated surveillance and action needed? *Euro Surveill*. 11:E060622.1. 10.2807/esw.11.25.02977-en 16819127

[B45] Steere A. C. Gross D. Meyer A. L. Huber B. T. (2001). Autoimmune mechanisms in antibiotic treatment-resistant Lyme arthritis. *J. Autoimmun.* 16 263–268. 10.1006/jaut.2000.0495 11334491

[B46] Tian H. Y. Zhou S. Dong L. Van Boeckel T. P. Cui Y. J. Wu Y. (2015). Avian influenza H5N1 viral and bird migration networks in Asia. *Proc. Natl. Acad. Sci. U.S.A.* 112 172–177. 10.1073/pnas.1405216112 25535385 PMC4291667

[B47] Tuchocka A. (2002). Arthritis in the course of Lyme disease. *Nowa Klin.* 9 1222–1227.

[B48] Tugwell P. (1997). Laboratory evaluation in the diagnosis of Lyme disease. *Ann. Intern. Med.* 127 1109–1123. 10.7326/0003-4819-127-12-199712150-00011 9412316

[B49] Wasiluk A. Zalewska-Szajda B. Waszkiewicz N. Kȩpka A. Szajda D. S. Wojewódzka-Żeleźniakowicz M. (2011). Lyme disease: etiology, pathogenesis, clinical courses, diagnostics and treatment. *Prog. Health Sci.* 1 179–186.

[B50] Watts N. Adger W. N. Agnolucci P. Costello A. (2015). Health and climate change: policy responses to protect public health. *Lancet* 386 1861–1914. 26111439 10.1016/S0140-6736(15)60854-6

[B51] Zajkowska J. Hermanowska–Szpakowicz T. (2002). New aspects of the pathogenesis of Lyme disease. *Przegl. Epidemiol*. 56(Suppl. 1) 57–67. 12194230

[B52] Zajkowska J. M. Hermanowska–Szpakowicz T. Pancewicz S. A. Kondrusik M. (2000). Selected aspects of immuno-patho-genesis in Lyme disease. *Pol. Merkur. Lekarski*. 9 579–583. 11081331

